# Supplementary figures and images for: Metabolomic Profiling of Female Mink Serum during Early to Mid-Pregnancy to Reveal Metabolite Changes
Source: Genes (Basel). 2023 Sep 4;14(9):1759. doi: 10.3390/genes14091759 (PMC10531253; doi:10.3390/genes14091759)

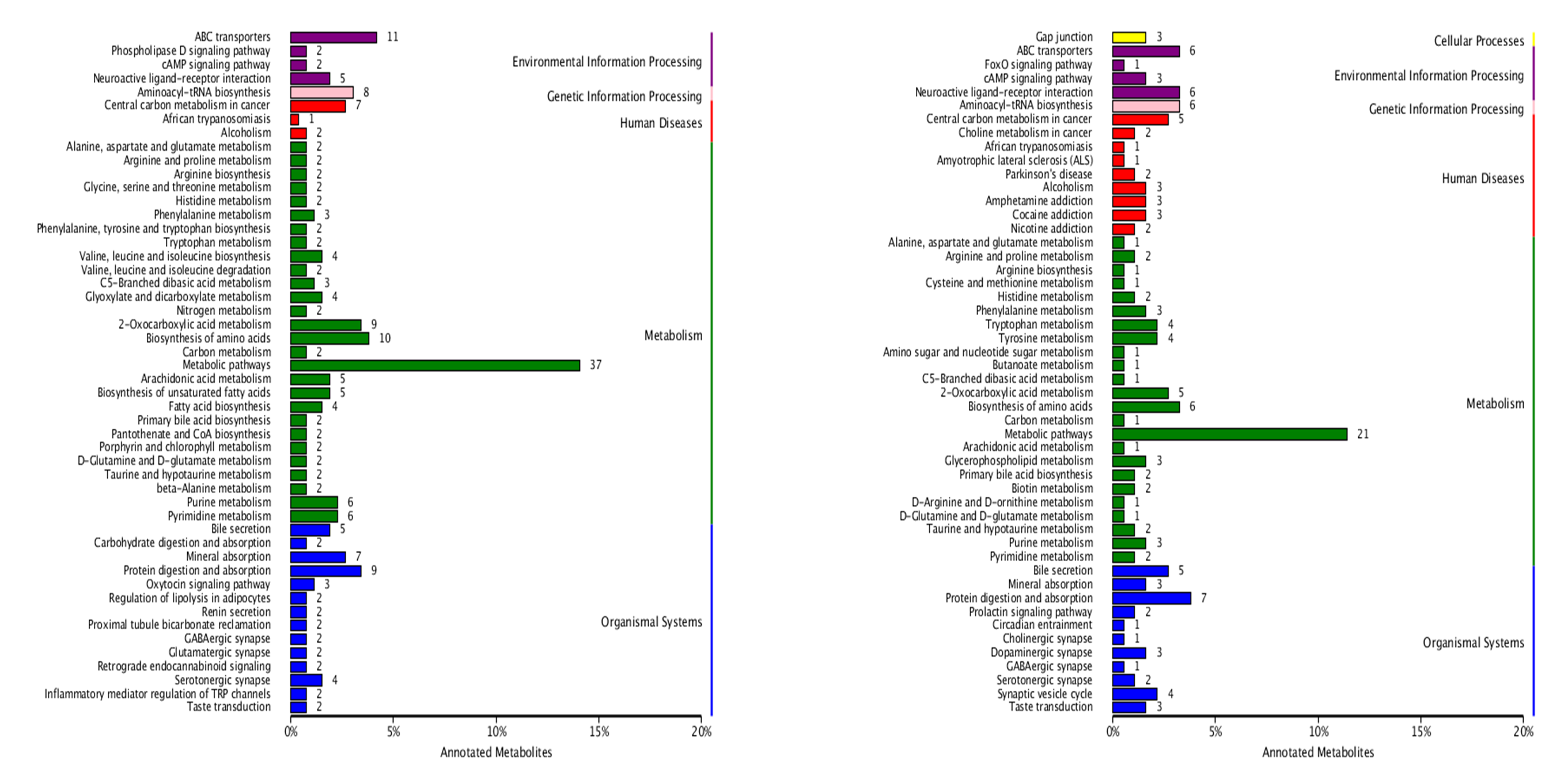

Supplement: Supplementary file 1 [file genes-14-01759-s001.zip › supplementary file-10-D1_vs_D8 Classification of pathway.tif]

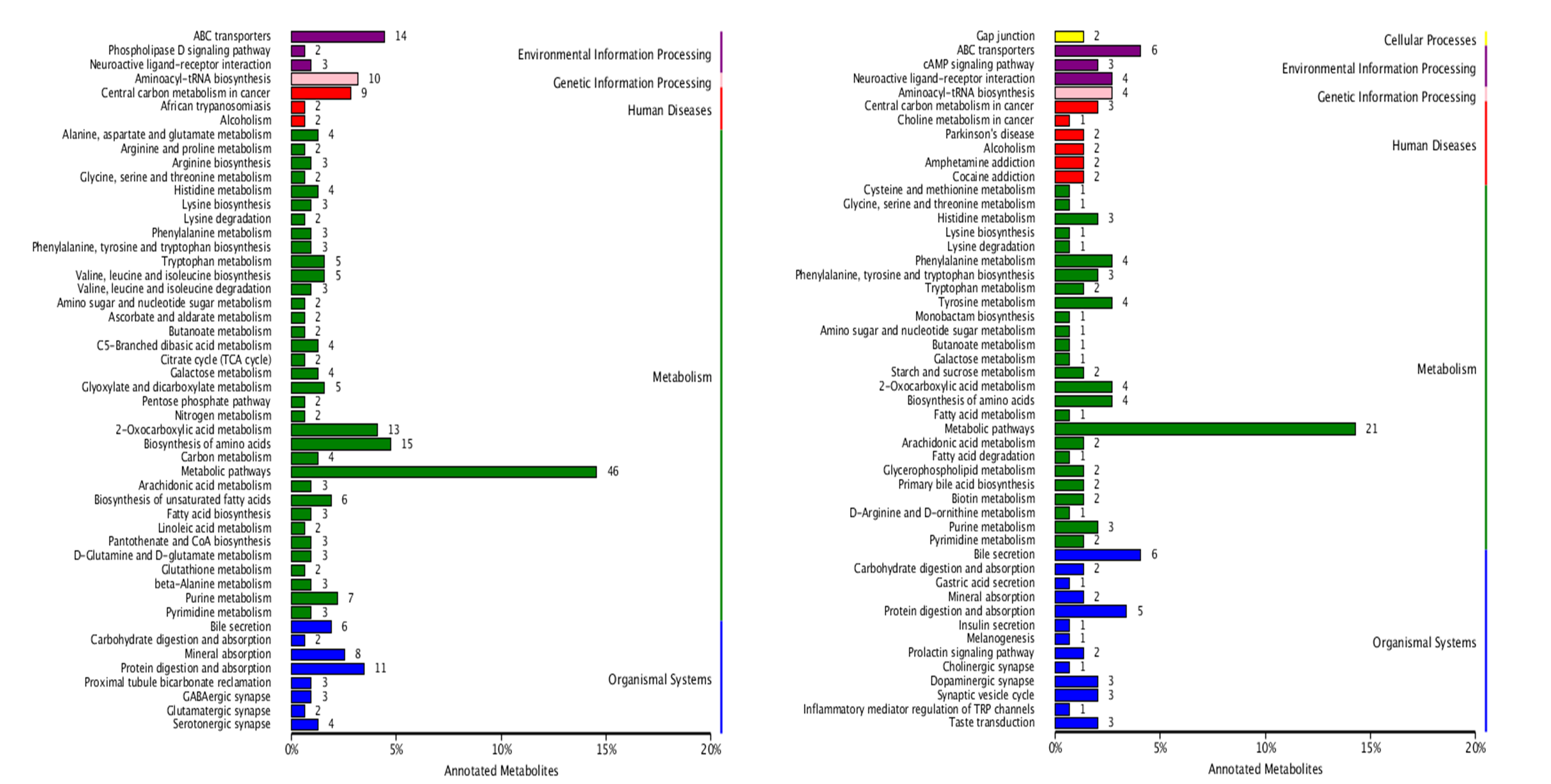

Supplement: Supplementary file 1 [file genes-14-01759-s001.zip › supplementary file-11-D1_vs_D15 Classification of pathway.tif]

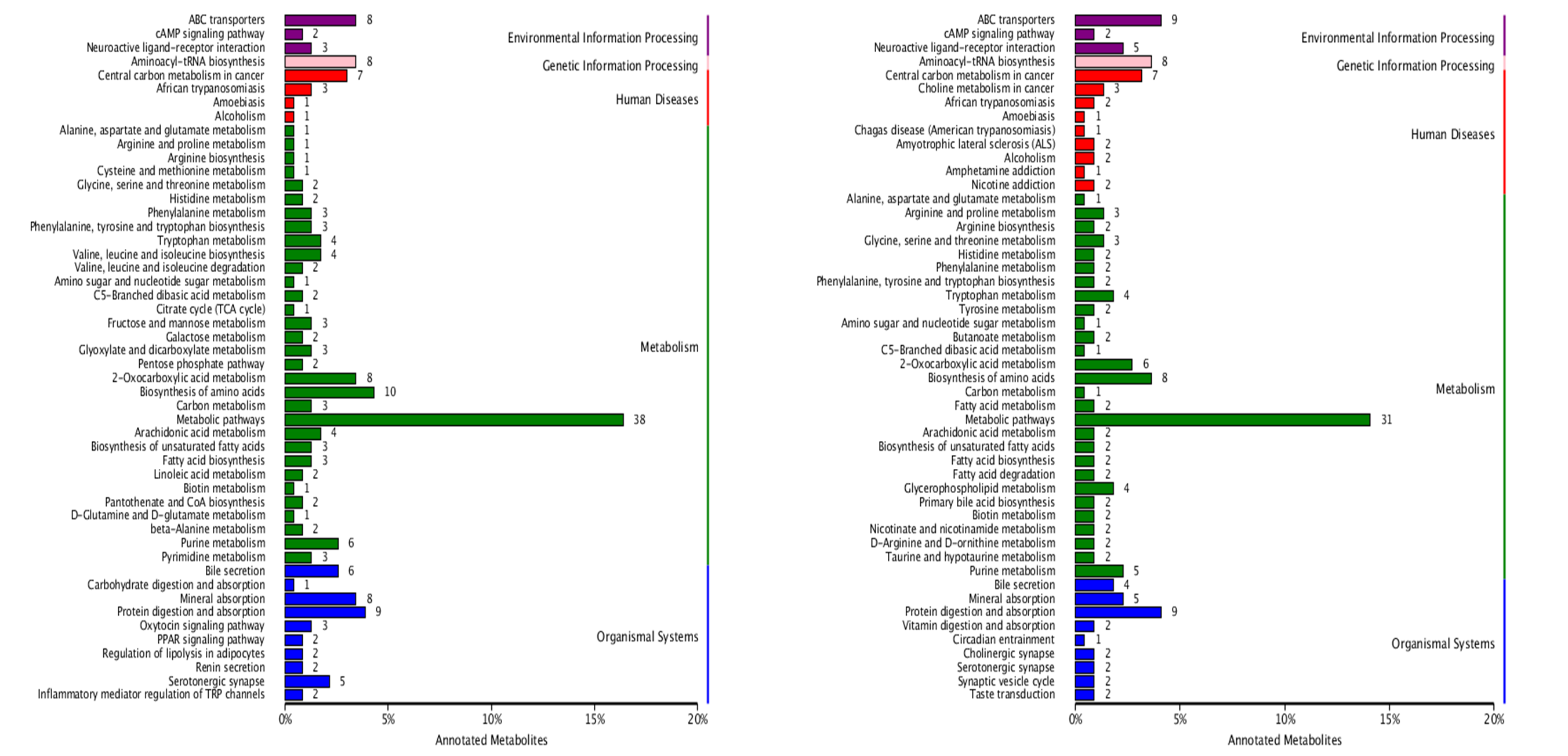

Supplement: Supplementary file 1 [file genes-14-01759-s001.zip › supplementary file-12-D1_vs_D22 Classification of pathway.tif]

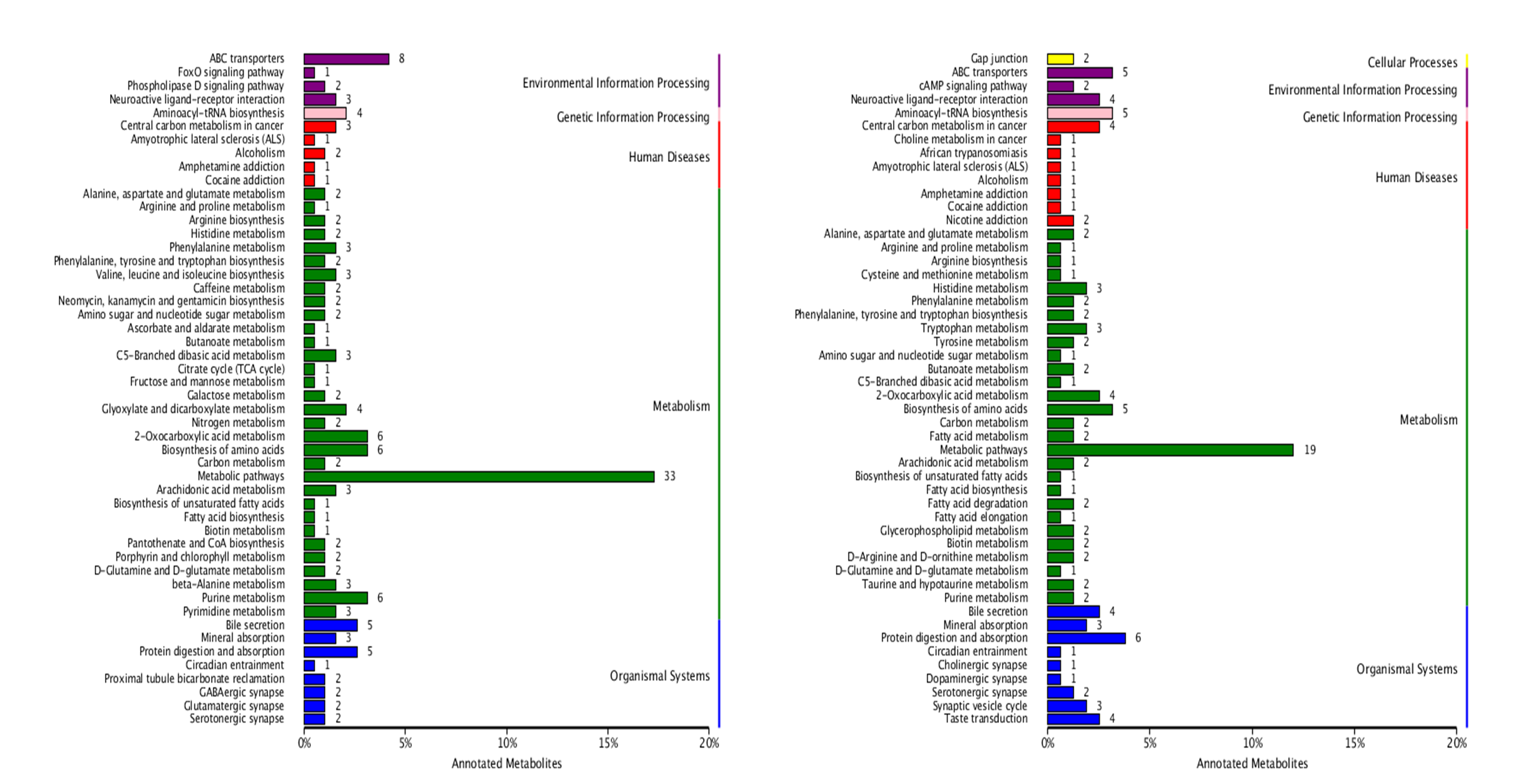

Supplement: Supplementary file 1 [file genes-14-01759-s001.zip › supplementary file-13-D1_vs_D29 Classification of pathway.tif]
